# Application of eye and hand interventions in brain magnetic resonance imaging of young children

**DOI:** 10.1016/j.heliyon.2024.e35613

**Published:** 2024-08-02

**Authors:** Qiying Ran, Xi Chen, Xiang Li, Ling He, Ke Zhang, Shilong Tang

**Affiliations:** a Department of Radiology Children's Hospital of Chongqing Medical University, National Clinical Research Center for Child Health and Disorders, Ministry of Education Key Laboratory of Child Development and Disorders, Chongqing Key Laboratory of Child Neurodevelopment and Cognitive Disorders, Chongqing, 400014, China; b Department of Equipment Children's Hospital of Chongqing Medical University, Chongqing, 400014, China

**Keywords:** Brain, MRI, Children, Eye, Hand

## Abstract

**Objective:**

To explore the feasibility of eye and hand interventions in young children during brain magnetic resonance imaging (MRI).

**Methods:**

A total of 414 4- to 6-year-old children who underwent brain MRI at our hospital were randomly divided into 4 groups: the routine posture group (n = 105), eye mask group (n = 102), fixed hand apron group (n = 108), and eye mask and fixed hand apron group (n = 99). All the children underwent brain MRI when they were awake (without using sedatives). The success rate of brain MRI and the quality of brain MR images were compared among the four groups.

**Results:**

The success rate of brain MRI was the highest in the eye mask and fixed hand apron group (94.9 %), followed by the eye mask group (85.3 %) (P < 0.05). The brain MR image quality was the best for children wearing eye masks and fixed hand aprons (5 points, 69 patients), followed by those wearing eye masks (5 points, 53 patients) (P < 0.05).

**Conclusion:**

When children undergo brain MRI, simultaneous eye and hand interventions can greatly improve the success rate of the examination and the quality of MR images. This study protocol was registered at the Chinese clinical trial registry (ChiCTR2100050248).

## 1 Background

Magnetic resonance imaging (MRI) is free of ionizing radiation and is one of the preferred methods for determining whether brain development and morphological structure in children are abnormal [[1], [2], [3]]. However, due to the active nature and poor self-control of young children, the success rate of brain MRI is relatively low when they are awake [[4], [5], [6], [7]].

When brain MRI examination cannot be completed in young children who are awake, drug sedation is often used. However, some parents refuse the examination because they are worried about the negative effects of drugs on their children. In children who refuse examination, a clinical diagnosis can only be made based on clinical manifestations, which may lead to inaccurate early diagnosis. This hinders early treatment and intervention in children, leading to disease progression [[8], [9], [10], [11]].

In previous studies, researchers have used many methods to improve the success rate of brain MRI in young children. For example, Carter AJ reported [12] that simulating MRI can improve the success rate of children's examinations, and Lindsay Woods-Frohlich reported [13] increased success with training children to reduce activity. However, there are still 47–61 % of children who cannot remain completely still during MRI due to fear or a lack of self-control, which ultimately causes MRI examination failure, in these children who failed the examination, approximately 83 % of children were aged 4–6 years old [[12], [13], [14], [15]]. In most of these studies, research focused on training children's ability to exercise self-control before the exam, and few researchers have studied “whether hand-eye interference in children can improve the success rate of MRI examinations.”

In our study, 4- to 6-year-old children without mental abnormalities underwent brain MRI while awake (without sedation) wearing eye masks and fixed hand aprons. We focused on the effectiveness and security of this intervention.

## 2 Materials and methods

### 2.1 Study participants

From October 2021 to July 2022, we prospectively recruited 414 children aged 4–6 years who needed brain MRI at the Affiliated Children's Hospital of Chongqing Medical University. The participants were randomly divided into 4 groups: the routine posture group (n = 105), eye mask group (n = 102), fixed hand apron group (n = 108) and eye mask and fixed hand apron group (n = 99) (Table 1).

**Table 1:** **Patient information** (Conventional posture group, n = 105; Eye mask group, n = 102; Fixed hand apron group, n = 108; Eye mask and fixed hand apron group, n = 99).

Group	Age	BMI	Scan time	Male to female ratio
Conventional posture group	4.51 ± 0.28	15.08 ± 0.58	6.47 ± 0.27	53:52
Eye mask group	4.49 ± 0.27	15.01 ± 0.55	6.53 ± 0.28	52:50
Fixed hand apron group	4.51 ± 0.29	15.02 ± 0.57	6.46 ± 0.30	55:53
Eye mask and fixed hand apron group	4.47 ± 0.29	14.99 ± 0.64	6.48 ± 0.29	44:55
degree of freedom	3	3	3	3
F value/χ^2^	0.297	0.449	1.529	1.221
P value	0.828	0.718	0.206	0.748

BMI= Body mass index.

The inclusion criteria for children in the study were as follows: their body mass index (BMI) was between 14 and 16, all children had MRI scans due to headache or dizziness, and their doctors were trying to rule out intracranial lesions. The patients had no history of head trauma, no functional diseases of the nervous system, no concomitant diseases of other organs, no other diseases that might affect brain function, and no abnormalities in routine magnetic resonance imaging of the head.

**Fig. 1:**
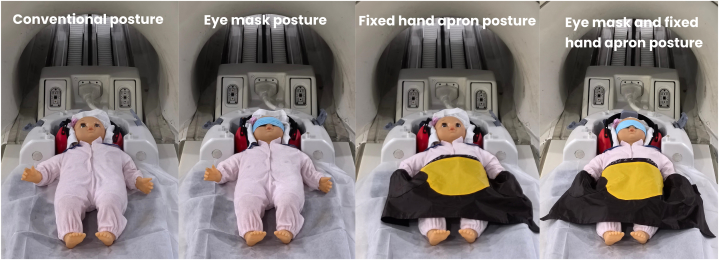
Schematic diagram of children's examination position.

In the routine posture group, the children lay on the examination bed in the supine position, with their hands flat on both sides of the body and their eyes slightly closed. In the eye mask group, the children lay on the examination bed in the supine position with their hands flat on both sides of the body and their eyes covered with a simple eye mask. In the fixed hand apron group, the children lay on the examination bed, in the supine position, with their hands flat in a special abdominal apron and their eyes slightly closed. In the eye mask and fixed hand apron group, the children lay flat on the examination bed in the supine position, with their hands flat in a special abdominal apron and their eyes covered with a simple eye mask (Fig. 1).

### 2.2 Study procedures

The children and their parents were prepared prior to the MRI (e.g., removing metal items from the patient's body and informing them that the MRI was noisy). During the examination, the children wore soundproof ear plugs and a headset to enhance the sound insulation effect. All the children were undergoing an MRI examination for the first time and had no previous MRI experience. All the children were examined between 3 and 6 p.m. and did not sleep for 6–8 h before the exam. All patients were psychologically advised (for example, the examination environment was described before the examination, and they were told that there would be considerable noise during the examination and that they needed to remain still during the examination).

All the data were collected in the Magnetic Resonance Room of the Children's Hospital of Chongqing Medical University using a GE Discover MR750 3.0T MR scanner with 8-channel head and neck coils, 5 scanning sequences (Sag T2, OAx T2, T1 FLAIR, T2 FLAIR, and DWI) and a scan time of 5–7 min. All the children were examined when they were awake (without using sedatives), and the success of the examination was recorded.

The quality evaluation criteria for brain MR images were as follows: 5 points: good image quality, very high signal-to-noise ratio (SNR), and no artifacts; 4 points: good image quality, high signal-to-noise ratio, no artifacts, and slight defects that do not affect image diagnosis; 3 points: good image quality, high signal-to-noise ratio, and slight motion artifacts that do not affect image diagnosis; 2 points: good image quality, high signal-to-noise ratio, and motion artifacts affecting image diagnosis; 1 point: poor image quality, poor signal-to-noise ratio, and artifacts, resulting in an inability to diagnose [[16], [17], [18], [19], [20]].

Two physicians with seven years of working experience rated the image quality. If there was any disagreement in the evaluation results, a third chief physician made a decision. All physicians rated the image quality without knowing the patient's information (gender, age, group, etc.). None of the physicians were at the examination site when evaluating the image quality. The MRI technician transmitted the images to the physician's workstation in real time through the internet, and the physician evaluated the image quality. If the image quality evaluation score was 3 points or more, the image quality met the diagnostic requirements, and the examination was successful. If the image quality evaluation score was less than 3 points, the image quality did not meet the diagnostic requirements. The MRI technician repeated the scan once after communicating with the patient and their parents; if the image quality score was still less than 3 points, the examination was considered to have failed.

During the study, the MRI staff, the patient, and the patient's parents were all aware of the patient's randomly assigned study group. The image quality evaluation physicians determined whether the image quality met the diagnostic requirements, whether the examination was successful or failed, and whether the child needed to be rescanned. To prevent personal factors from influencing repeated scanning decisions, the image quality evaluation physicians did not know the children's information (age, sex, group, etc.).

The evaluation criteria for participant comfort were as follows: 5 points: the test comfort was very good; there was no discomfort, and the test was successfully completed; 4 points: the test comfort was good; there was some discomfort, but it did not affect the test, and the test was successfully completed; 3 points: the test comfort was good; there was some discomfort that could be overcome and may have affected the test, and the test continued but failed; 2 points: the test comfort was poor; there was discomfort that could not be overcome, and the test could not continue and was considered a failure. (All participants' comfort evaluations were completed by the children themselves, with the doctor asking questions and the children answering.)

### 2.3 Statistical analysis

SPSS 26.0 statistical software was used for statistical analysis. All quantitative data (age, BMI, scan time, etc.) were normally distributed. The average standard deviation was calculated, and multigroup comparison analysis was performed. There were significant differences in the results, and correction of P-values using the Bonferroni method in multiple tests. The qualitative data (sex, image quality score, participant comfort score, MRI examination completion status, etc.) are described using frequency, and a chi-square test was used for intergroup comparisons. There were significant differences in the results, and correction of P-values using the Bonferroni method in multiple tests. P < 0.05 was considered to indicate statistical significance. All P-values in the reports are corrected.

## 3 Results

**Comparison of children's basic information** There were no significant differences in age, sex, BMI or scanning time among the four groups of children (p > 0.05) (Table 1).

**Fig. 2:**
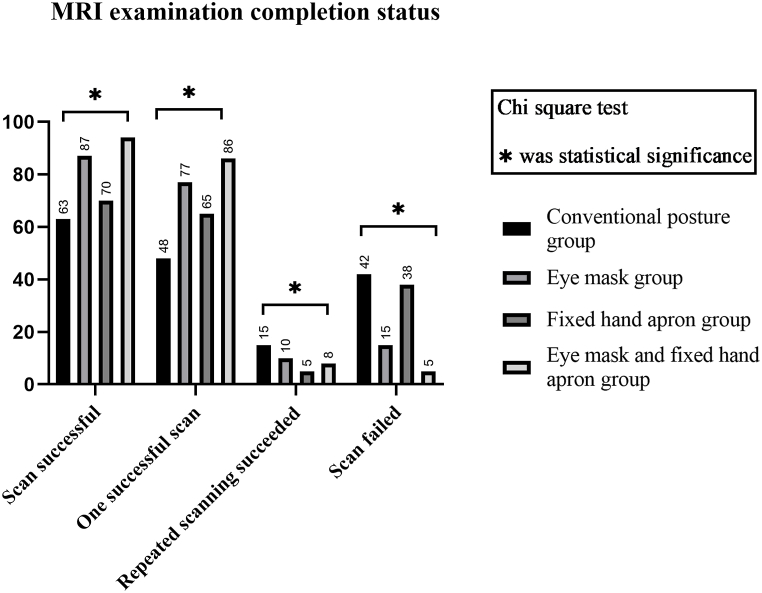
Bar chart of magnetic resonance examination completion status.

**Comparison of the completion of brain MRI examination** The eye mask and fixed hand apron group had the highest examination success rate (94.9 %) and one-time examination success rate (86.9 %), followed by the eye mask group. The lowest examination success rate was found in the routine posture group (60.0 %), and the difference was statistically significant (p < 0.001). There was no statistically significant difference in the number of children who were asleep after the examination (p = 0.854) (Table 2, Fig. 2).

**Table 2:** **MRI examination completion status** (Conventional posture group, n = 105; Eye mask group, n = 102; Fixed hand apron group, n = 108; Eye mask and fixed hand apron group, n = 99).

Group	Scan successful	One successful scan	Repeated scanning succeeded	Scan failed	Scan success rate (%)	One time scanning success rate (%)	Number of children fall sleep during MRI scan
Conventional posture group	63	48	15	42	60	45.7	5
Eye mask group	87	77	10	15	85.3	75.5	6
Fixed hand apron group	70	65	5	38	64.8	60.2	4
Eye mask and fixed hand apron group	94	86	8	5	94.9	86.9	6
degree of freedom	3	3	3	3	3	3	3
χ^2^ value	46.256	44.540	5.631	46.256	46.256	44.540	0.781
P value	<0.001	<0.001	0.031	<0.001	<0.001	<0.001	0.854

**Comparison of failed MRI examinations** Among the children in whom MRI scans failed, there was no significant difference in age (p = 0.39), and the number of boys was significantly greater than the number of girls (p < 0.001). Among all MR sequences, the T1 FLAIR sequence failed most often, and the difference was statistically significant (p < 0.001) (Table 3).

**Table 3:** **Scan failed children related information** (Conventional posture group, n = 105; Eye mask group, n = 102; Fixed hand apron group, n = 108; Eye mask and fixed hand apron group, n = 99).

Group	Scan failed						
	Male to female ratio	Age	Scan failed sequence				
			Sag T2	OAx T2	T1 Flair	T2 Flair	DWI
Conventional posture group	29:13	4.50 ± 0.29	0	1	36	5	0
Eye mask group	8:7	4.54 ± 0.25	0	0	9	6	0
Fixed hand apron group	23:15	4.49 ± 0.32	1	4	21	12	0
Eye mask and fixed hand apron group	3:2	4.25 ± 0.37	0	0	3	2	0
degree of freedom	3	3	3	3	3	3	–
F value/χ^2^	16.855	1.014	2.814	8.154	41.841	8.021	–
P value	<0.001	0.39	0.841	0.043	<0.001	0.046	–

**Fig. 3:**
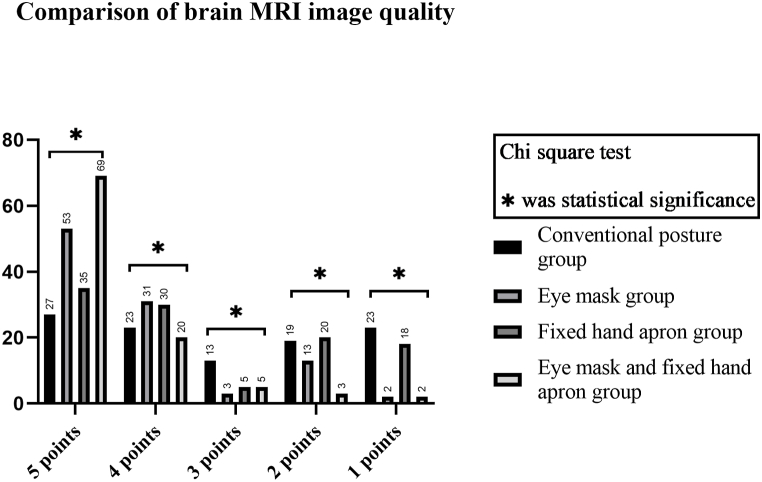
Bar chart of MRI brain image quality comparison.

**Comparison of MR image quality** The brain MR image quality was highest in the eye mask and fixed hand apron group (5 points, 69 patients) followed by the eye mask group (5 points, 53 patients), and the difference was statistically significant (p < 0.001) (Table 4, Fig. 3).

**Table 4:** **Comparison of brain MRI image quality** (Conventional posture group, n = 105; Eye mask group, n = 102; Fixed hand apron group, n = 108; Eye mask and fixed hand apron group, n = 99).

Group	5 points	4 points	3 points	2 points	1 points
Conventional posture group	27	23	13	19	23
Eye mask group	53	31	3	13	2
Fixed hand apron group	35	30	5	20	18
Eye mask and fixed hand apron group	69	20	5	3	2
degree of freedom	3	3	3	3	3
χ^2^ value	49.158	3.094	9.326	13.740	33.303
P value	<0.001	0.377	0.025	0.003	<0.001

**Participant comfort level** The participant comfort level was highest in the eye mask and fixed hand apron group (4 points, 79 patients). The second highest comfort level was observed in the eye mask group (4 points, 71 patients), and the difference was statistically significant (p < 0.001) (Table 5).

**Table 5:** **Participant comfort level** (Conventional posture group, n = 105; Eye mask group, n = 102; Fixed hand apron group, n = 108; Eye mask and fixed hand apron group, n = 99).

Group	5 points	4 points	3 points	2 points	1 points
Conventional posture group	5	43	15	11	31
Eye mask group	6	71	10	9	6
Fixed hand apron group	5	60	5	13	25
Eye mask and fixed hand apron group	7	79	8	2	3
degree of freedom	3	3	3	3	3
χ^2^ value	0.756	37.002	6.157	7.659	39.122
P value	0.860	<0.001	0.104	0.054	<0.001

## 4 Discussion

With the extensive use of MRI in the diagnosis of childhood diseases, the number of children requiring sedation continues to increase. Parents' awareness of the risks and costs of sedation is also increasing, causing some parents to refuse MRI. In the past, radiologists used various methods to test some children successfully, but many tests still failed, especially for preschool children [[21], [22], [23], [24], [25]]. This study used eye masks and fixed hand aprons to interfere with the hands and eyes during MRI examinations in 4-to 6-year-olds, which greatly increased the success rate of head MRI examinations in children in this age group.

The conventional posture group had the largest number of rescan and scan failure cases (57 cases) and the lowest one-time success rate (45.20 %). This may be because the children are young and did not have effective restrictions on their eyes and hands. As a result, children often involuntarily open their eyes, rotate their heads, and move their hands [[22], [23], [24], [25]].

In this study, the success rate of examination and the quality of brain MR images of children in the three study groups (eye mask group, fixed hand apron group, eye mask and fixed hand apron group) were greater than those in the routine posture group. In the eye mask and fixed hand apron group, participants wore eye masks and used fixed hand aprons to interfere with their eyes and hands at the same time. This approach achieved an excellent examination success rate (94.9 %). Some parents believed that their children could not tolerate the process and would want to refuse the examination when they were exposed to the MRI environment. However, after intervention measures, such as wearing an eye mask and using a fixed hand apron, the child successfully completed the MRI examination, which surprised the parents. Blinking or eye movement can lead to motion artifacts in MR images. By wearing an eye mask, image motion artifacts caused by eye opening, blinking or movement can be reduced or avoided. This approach not only minimizes the artifacts caused by the random movement of the head in the head coils when the children open their eyes but also reduces or prevents the children from becoming increasingly nervous about the narrow magnets and preventing them from adhering to the MRI process. Wearing eye masks reduces children's fear, encourages them to remain quiet, and makes it easier for them to cooperate with the examination [22,23]. In addition, the hands are fixed in the abdominal apron pocket in a natural and comfortable position, which makes the child's hands feel more secure. This reduces tension and fear and encourages the child to be quiet. This approach also avoids failure of the examination caused by the random movement of the head due to random movement of the hands and prevents the children who do not cooperate with the examination from pinching their hands when the bed is moving, ensuring their safety.

Comparing the eye mask group and the fixed hand apron group, the examination success rate and brain MR image quality were significantly greater in the eye mask group. This phenomenon may be because wearing an eye mask can help children easily keep their eyes closed, reducing the movement artifacts caused by eye movement or blinking on MR images.

The present study revealed that there were fewer children in the all group who slept after the scan, the difference was not statistically significant (p = 0.854). This may be because, at the time of the examination, the children were in an unfamiliar environment with loud noise, and although the children did not sleep for a long time before the test, it was not easy for them to sleep well after the scan.

The children in this study were all 4–6 years old because, prior to this study, we found that some children in this age group could successfully complete a head MRI examination while awake (without using sedatives). Under the age of 4, most children cannot complete a head MRI examination while awake, while only a very small number of children older than 6 years cannot complete a head MRI examination while awake. Therefore, we chose only 4- to 6-year-old children; other age groups will be included in future studies. Our preliminary study revealed that children aged less than 4 years had fewer successful magnetic resonance examinations, which is inconsistent with the findings of Antonov NK [22]. It is possible that our pre-MRI preparations were not as detailed as those of the previous study (for example, scanning during nap times). Therefore, children under age 4 will be the focus of our next study.

This study showed that the MRI examination failed in more male children than female children. It is possible that in children in this age group, female children have earlier brain development than male children; therefore, female children are more likely to cooperate with MRI tests [[26], [27], [28]]. The researchers also found that the T1 FLAIR test had the highest failure rate. It is possible that this sequence requires a higher degree of cooperation from children than other sequences. A slight movement of the examiner may cause this sequence to fail.

After the test was completed, we invited the children to rate their comfort with the test. The children who failed the test all scored their comfort below 3 points. These children generally feared the unfamiliar narrow environment and the noise, which ultimately led to a lower comfort score. Among the children who were successfully tested, those in the eye mask and fixed hand apron group had higher comfort scores. The fixed hand apron gave the child a comfortable place to rest their hands, so the comfort score was the highest. The conventional posture group had the lowest comfort score. Children in this group experienced loud noise during the examination, and placing their hands on both sides of their body to avoid manual interference and seeing the narrow space during MRI examination without eye masks to cover their eyes caused tension. The comfort score of the eye mask group was greater than that of the fixed hand apron group, possibly because the unfamiliar environment made the children more nervous than having to keep their hands in place. The study also found that in the 4 groups of children, there were few children with a comfort score of 5, and the difference among the groups was not statistically significant (P = 0.860). Children generally responded to the noise during the MRI examination, which affected their comfort level.

In our previous preliminary study, we found that, among children of the same age group, children with high BMI had a greater test success rate than children with low BMI (the reason may be that children with high BMI are more mature than children with low BMI) [[29], [30], [31]]. To rule out the effects of BMI that are too high or too low on the success rate of MRI, we selected children in this age group with a BMI in the normal range (16–18).

In the present study, the examination success rates of children in the eye mask group and the eye mask and fixed hand group were significantly greater than those in the remaining two groups, and the success rate in the eye mask group was slightly lower than that in the eye mask and fixed hand group but significantly greater than that in the fixed hand apron group. These findings indicate that eye masks can significantly improve the success rate of MRI. The reason for this result is that eye masks can not only reduce head MR image motion artifacts caused by eyes moving or blinking but also decrease children's fears triggered by environmental changes. Therefore, in the absence of hand aprons, disposable masks can be made into simple eye masks to improve the success rate of MRI in young children.

The advantages of this study include the following: a large sample size, forward-looking design, fixed hand aprons that can be reused, and simple and convenient eye mask collection. This study also has several limitations. For example, it was impossible to blind the children, parents, or staff conducting the scans to the group to which each participant was assigned. The simple eye masks used in the study were all modified after removing metal strips from the masks worn by the children. The materials of these simple eye masks may have been different: they may have been slightly softer or slightly harder, or they may have provided stronger or weaker protection from the light. Some children complained of discomfort. With the unified use of soft and strong anti-light material in eye masks, the effect will improve. In this study, all the children routinely underwent sleep deprivation and psychological counseling. There were differences in the duration of sleep deprivation and compliance of children and parents. There were also individual differences in the daily sleep needs of each child. These factors may have led to differences in the results of the study.

In summary, in this study, brain magnetic resonance imaging with eye and hand interventions was performed in 4- to 6-year-old children with normal intelligence. This approach can greatly improve the success rate of MRI and image quality in children and prevent pain and complications caused by drug sedation. It also provides a new possible direction for brain MRI in younger children who are awake (without sedatives). This research method has the advantages of simple consumables, convenient operation and no side effects for children. It has important clinical significance and obvious social benefits, so it is worth popularizing and applying.

## Funding statement

This study protocol was supported by grants from National Clinical Research Center for Child Health and Disorders (Children's Hospital of 10.13039/501100004374Chongqing Medical University, Chongqing, China) (grant number NCRCCHD-2021-YP-07) and 10.13039/501100007957Chongqing Municipal Education Commission （Chongqing, China） (NO. KJQN202000425).

## Conflict of interest disclosure

There is no conflicts of interest in this study.

## Ethics approval statement and patient consent statement

This study was approved by the Ethics Committee of Children's Hospital Affiliated to Chongqing Medical University (NO.2021–275), and the family members of the study children signed an informed consent form before the examination. All methods were performed in accordance with the relevant guidelines and regulations.

### Clinical trial registration

This study protocol was registered at the Chinese clinical trial registry (ChiCTR2100050248).

## Trial registration

This study protocol was registered at the Chinese clinical trial registry (NO. ChiCTR2100050248; August 24, 2021).

## Data availability statement

Qiying Ran and Shilong Tang had control of the study data.

All data is available (https://figshare.com/s/b2b40f17add6dd808b25).

## Ethics declarations

This study was reviewed and approved by [Human Ethics Committee of the Children's Hospital of Chongqing Medical University], with the approval number: [No. 2021–275]. All participants/patients (or their proxies/legal guardians) written informed consent was obtained in the study. All participants/patients (or their proxies/legal guardians) provided informed consent for the publication of their anonymised case details and images.

## CRediT authorship contribution statement

**Qiying Ran:** Writing – original draft, Data curation. **Xi Chen:** Software, Data curation. **Xiang Li:** Resources, Data curation. **Ling He:** Writing – review & editing. **Ke Zhang:** Resources, Data curation. **Shilong Tang:** Writing – review & editing, Funding acquisition, Conceptualization.

## Declaration of competing interest

The authors declare that they have no known competing financial interests or personal relationships that could have appeared to influence the work reported in this paper.

## References

[1] Vijayakumar N., Youssef G., Allen N.B., Anderson V., Efron D., Mundy L., Patton G., Simmons J.G., Silk T., Whittle S. (2021). The effects of puberty and its hormones on subcortical brain development. Compr Psychoneuroendocrinol.

[2] Han X., Wei L., Sun Y., Hu Y., Wang Y., Ding W., Wang Z., Jiang W., Wang H., Zhou Y. (2021). MRI-based radiomic machine-learning model may accurately distinguish between subjects with internet gaming disorder and healthy controls. Brain Sci..

[3] Rohilla S., Duhan A., Bala K., Kaushik J.S. (2021). Brain perfusion, hippocampal volumetric, and diffusion-weighted imaging findings in children with prolonged febrile seizures and focal febrile seizures. J. Pediatr. Neurosci..

[4] Tang Shilong, Zhang Guanping, Ran Qiying, Liu Xianfan, Pan Zhengxia, He Ling (2022). Quantitative susceptibility mapping shows lower brain iron content in children with attention-deficit hyperactivity disorder. Hum. Brain Mapp..

[5] Tang Shilong, Xu Ye, Liu Xianfan, Chen Zhuo, Zhou Yu, Nie Lisha, He Ling (2021). Quantitative susceptibility mapping shows lower brain iron content in children with autism. Eur. Radiol..

[6] Tang S., Nie L., Liu X., Chen Z., Zhou Y., Pan Z., He L. (2021). Application of quantitative magnetic resonance imaging in the diagnosis of autism in children. Front. Med..

[7] Greene D.J., Koller J.M., Hampton J.M., Wesevich V., Van A.N., Nguyen A.L., Hoyt C.R., McIntyre L., Earl E.A., Klein R.L., Shimony J.S., Petersen S.E., Schlaggar B.L., Fair D.A., Dosenbach N.U.F. (2018). Behavioral interventions for reducing head motion during MRI scans in children. Neuroimage.

[8] Tang Shilong, Liu Xianfan, Ran Qiying, Nie Lisha, Wu Lan, Pan Zhengxia, He Ling (2022). Application of three-dimensional arterial spin labeling (3D-ASL) perfusion imaging in the brain of children with autism. Front. Neurol..

[9] Peng S.J., Hsieh K.L., Lin Y.K., Tsai M.L., Wong T.T., Chang H. (2021). Febrile seizures reduce hippocampal subfield volumes but not cortical thickness in children with focal onset seizures. Epilepsy Res..

[10] Moon J.U., Han J.Y. (2021). Effectiveness of chloral hydrate on brain MRI in children with developmental delay/intellectual disability comparing with normal intelligence: single tertiary center experience. Children.

[11] Shen F., Zhang Q., Xu Y., Wang X., Xia J., Chen C., Liu H., Zhang Y. (2021). Effect of intranasal dexmedetomidine or midazolam for premedication on the occurrence of respiratory adverse events in children undergoing tonsillectomy and adenoidectomy: a randomized clinical trial. JAMA Netw. Open.

[12] Carter A.J., Greer M.L., Gray S.E., Ware R.S. (2010). Mock MRI: reducing the need for anaesthesia in children. Pediatr. Radiol..

[13] Szeszak S., Man R., Love A., Langmack G., Wharrad H., Dineen R.A. (2016). Animated educational video to prepare children for MRI without sedation: evaluation of the appeal and value. Pediatr. Radiol..

[14] Runge S.B., Christensen N.L., Jensen K., Jensen I.E. (2018). Children centered care: minimizing the need for anesthesia with a multi-faceted concept for MRI in children aged 4-6. Eur. J. Radiol..

[15] Heales C.J., Lloyd E. (2022). Play simulation for children in magnetic resonance imaging. J. Med. Imag. Radiat. Sci..

[16] Nikam R.M., Yue X., Kaur G., Kandula V., Khair A., Kecskemethy H.H., Averill L.W., Langhans S.A. (2022). Advanced neuroimaging approaches to pediatric brain tumors. Cancers.

[17] Okudzhava L., Heldmann M., Münte T.F. (2022). A systematic review of diffusion tensor imaging studies in obesity. Obes. Rev..

[18] Ilyka D., Johnson M.H., Lloyd-Fox S. (2021). Infant social interactions and brain development: a systematic review. Neurosci. Biobehav. Rev..

[19] Copeland A., Silver E., Korja R., Lehtola S.J., Merisaari H., Saukko E., Sinisalo S., Saunavaara J., Lähdesmäki T., Parkkola R., Nolvi S., Karlsson L., Karlsson H., Tuulari J.J. (2021). Infant and child MRI: a review of scanning procedures. Front. Neurosci..

[20] Yang J.Y., Yeh C.H., Poupon C., Calamante F. (2021). Diffusion MRI tractography for neurosurgery: the basics, current state, technical reliability and challenges. Phys. Med. Biol..

[21] Kurugol S., Seager C.M., Thaker H., Coll-Font J., Afacan O., Nichols R.C., Warfield S.K., Lee R.S., Chow J.S. (2020). Feed and wrap magnetic resonance urography provides anatomic and functional imaging in infants without anesthesia. J. Pediatr. Urol..

[22] Antonov N.K., Ruzal-Shapiro C.B., Morel K.D., Millar W.S., Kashyap S., Lauren C.T., Garzon M.C. (2017). Feed and wrap MRI technique in infants. Clin. Pediatr..

[23] Suzuki A., Yamaguchi R., Kim L., Kawahara T., Ishii-Takahashi A. (2023). Effectiveness of mock scanners and preparation programs for successful magnetic resonance imaging: a systematic review and meta-analysis. Pediatr. Radiol..

[24] hieba C., Frayne A., Walton M., Mah A., Benischek A., Dewey D., Lebel C. (2018). Factors associated with successful MRI scanning in unsedated young children. Front Pediatr.

[25] ajagopal A., Byars A., Schapiro M., Lee G.R., Holland S.K. (2014). Success rates for functional MR imaging in children. AJNR Am J Neuroradiol.

[26] Reiss A.L., Abrams M.T., Singer H.S., Ross J.L., Denckla M.B. (1996). Brain development, gender and IQ in children. A volumetric imaging study. Brain.

[27] Potter A., Dube S., Allgaier N., Loso H., Ivanova M., Barrios L.C., Bookheimer S., Chaarani B., Dumas J., Feldstein-Ewing S., Freedman E.G., Garavan H., Hoffman E., McGlade E., Robin L., Johns M.M. (2021). Early adolescent gender diversity and mental health in the Adolescent Brain Cognitive Development study. JCPP (J. Child Psychol. Psychiatry).

[28] Potter A.S., Dube S.L., Barrios L.C., Bookheimer S., Espinoza A., Feldstein Ewing S.W., Freedman E.G., Hoffman E.A., Ivanova M., Jefferys H., McGlade E.C., Tapert S.F., Johns M.M. (2022). Measurement of gender and sexuality in the adolescent brain cognitive development (ABCD) study. Dev Cogn Neurosci.

[29] Dennis E., Manza P., Volkow N.D. (2022). Socioeconomic status, BMI, and brain development in children. Transl. Psychiatry.

[30] Alosco M.L., Stanek K.M., Galioto R., Korgaonkar M.S., Grieve S.M., Brickman A.M., Spitznagel M.B., Gunstad J. (2014). Body mass index and brain structure in healthy children and adolescents. Int. J. Neurosci..

[31] Masterson T.D., Bobak C., Rapuano K.M., Shearrer G.E., Gilbert-Diamond D. (2019). Association between regional brain volumes and BMI z-score change over one year in children. PLoS One.

